# Supplementary material for: Myoglobin Concentration and Oxygen Stores in Different Functional Muscle Groups from Three Small Cetacean Species
Source: Animals (Basel). 2021 Feb 9;11(2):451. doi: 10.3390/ani11020451 (PMC7915992; doi:10.3390/ani11020451)
Supplement: Supplementary file 1 [file animals-11-00451-s001.pdf]

Supplementary materials

Table S1. Parameters of the calibration adjustment and absorbance values for known concentrations of standard horse Mb.

	Value	Error	p-value
**Intercept**	0.0182	0.00692081	0.018257
**Slope**	0.1124	0.003676638	1.27E-15
**Standard Deviation**	0.0217		
**Multiple R**	0.9915		
**N**	18		
**M**	9		
**Detection limit**	0.18471912	**mg/L**	**LD**
**Quantification limit**	0.6	**mg/L**	**LQ**

**Calibration curve data**			
**mg/ml**	538 nm	568nm	**A_{538} - A_{568}**
0	0.059	0.059	0.000
0	0.059	0.058	0.001
0	0.079	0.079	0.000
0.1	0.078	0.063	0.015
0.1	0.081	0.066	0.015
0.1	0.078	0.063	0.015
0.5	0.384	0.310	0.074
0.5	0.386	0.313	0.073
0.5	0.382	0.308	0.074
1	0.771	0.618	0.153
1	0.770	0.618	0.152
1	0.774	0.622	0.152
2	1.386	1.111	0.275
2	1.392	1.115	0.277
2	1.387	1.112	0.275
4	2.285	1.838	0.447
4	2.287	1.841	0.446
4	2.285	1.838	0.447

Table S2. Mb concentration raw values in g Mb · 100 g^{-1} muscle (mean ± *S.D.*) among the different muscles/muscle locations for the three cetacean species studied.

	***D. delphis* (*n*=4)**	***S. coeruleoalba* (*n*=3)**	***S. frontalis* (*n*=4)**
		**Mean ± *S.D.***	
Mastohumeralis	1.584 ± *0.444*	2.597 ± *0.690*	1.414 ± *0.095*
Dorsal scalenus	2.038 ± *0.392*	3.657 ± *0.559*	1.983 ± *0.500*
Sternohyoideus	1.615 ± *0.349*	2.091 ± *0.428*	1.298 ± *0.099*
**Epaxial axilla**	3.920 ± *0.798*	6.394 ± *0.249*	2.992 ± *0.249*
**Epaxial middle**	4.650 ± *0.740*	6.605 ± *0.778*	3.534 ± *0.268*
**Epaxial anus**	4.431 ± *0.943*	5.921 ± *0.605*	3.092 ± *0.353*
**Hypaxial middle**	4.729 ± *0.314*	7.005 ± *0.650*	3.412 ± *0.107*
**Hypaxial anus**	4.931 ± *0.705*	6.598 ± *0.682*	2.883 ± *0.163*
Rectus abdominis	4.576 ± *0.347*	6.385 ± *0.424*	3.242 ± *0.245*

Figure S1. Boxplot representing Mb concentration raw (real) values in g Mb · 100 g^{-1} muscle for the different muscles and muscle locations for the three species included in the current study (*S. coeruleoalba* in yellow, *D. delphis* in purple and *S. frontalis* in grey).

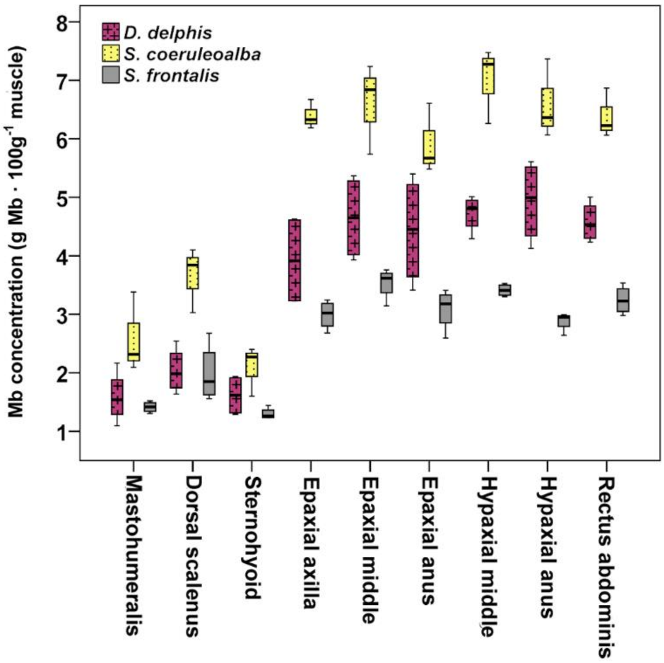

Table S3. Mb concentration raw values in g Mb · 100 g-1 muscle (mean ± *S.D.*) calculated using Reynafarje equation (1963) and the calibration curve for the individual *S. frontalis* CET 834.

	**Reynafarje equation**		**Calibration curve**	
	**Mean**	***S.D.***	**Mean**	***S.D.***
Mastohumeralis	1.303	*0.391*	1.329	*0.059*
Dorsal scalenus	1.689	*0.080*	1.915	*0.122*
Sternohyoideus	1.230	*0.046*	1.219	*0.069*
**Epaxial axilla**	2.920	*0.123*	3.781	*0.187*
**Epaxial middle**	3.760	*0.363*	5.054	*0.55*
**Epaxial anus**	3.410	*0.041*	4.524	*0.062*
**Hypaxial middle**	3.472	*0.068*	4.619	*0.104*
**Hypaxial anus**	2.993	*0.075*	3.8917	*0.114*
Rectus abdominis	2.977	*0.110*	3.8679	*0.167*

Table S4. O_2 stored (mL) (raw values) within the individuals' different functional muscle groups included in the present study.

		**O_2 stored within the different functional groups (mL)**						
		**Appendicular movement**	**Respiration**	**Feeding**	**Upstroke movement**	**Downstroke movement**	**Downstroke assistance**	**Total O_2**
D. delphis	**IFAW 14/044**	26.18	50.76	9.04	684.26	450.38	187.22	1407.84
	**IFAW 14/134**	104.81	145.23	16.21	1635.5	1013.96	390.46	3306.17
	**CET 745**	17.56	42.20	8.60	547.23	276.38	150.20	1042.17
	**CET 767**	9.14	119.02	18.28	1141.87	458.3	242.77	2050.46
S. coeruleoalba	**CET 748**	59.83	102.81	18.25	1450.57	661.69	272.03	2565.18
	**CET 750**	49.93	115.86	15.67	2144.72	1080.95	357.48	3764.61
	**CET 837**	44.57	88.27	13.65	1354.52	631.44	281.11	2413.56
S. frontalis	**CET 822**	25.91	36.66	7.44	714.72	348.32	201.03	1334.08
	**CET 829**	10.16	24.45	3.97	246.38	110.06	62.86	457.88
	**CET 830**	22.16	101.19	7.3	685.29	242.42	192.83	1251.19
	**CET 834**	18.09	53.59	5.87	700.33	303.67	168.00	1249.55
